# Hyperglycaemia, diabetes and risk of fragility fractures: observational and Mendelian randomisation studies

**DOI:** 10.1007/s00125-023-06054-8

**Published:** 2023-12-14

**Authors:** Frida Emanuelsson, Shoaib Afzal, Niklas R. Jørgensen, Børge G. Nordestgaard, Marianne Benn

**Affiliations:** 1 Department of Clinical Biochemistry, Copenhagen University Hospital Rigshospitalet, Centre of Diagnostic Investigation, Copenhagen, Denmark; 2 Department of Clinical Medicine, Faculty of Health and Medical Sciences, University of Copenhagen, Copenhagen, Denmark; 3 Department of Clinical Biochemistry, Copenhagen University Hospital Herlev and Gentofte, Herlev, Denmark; 4 The Copenhagen General Population Study, Copenhagen University Hospital Herlev and Gentofte, Herlev, Denmark; 5 Department of Clinical Biochemistry, Copenhagen University Hospital Rigshospitalet, Centre of Diagnostic Investigation, Glostrup, Denmark

**Keywords:** Fragility fracture, Hyperglycaemia, Mendelian randomisation, Type 1 diabetes, Type 2 diabetes

## Abstract

**Aims/hypothesis:**

Fragility fractures may be a complication of diabetes, partly caused by chronic hyperglycaemia. We hypothesised that: (1) individuals with hyperglycaemia and diabetes have increased risk of fragility fracture; (2) hyperglycaemia is causally associated with increased risk of fragility fracture; and (3) diabetes and fragility fracture jointly associate with the highest risk of all-cause mortality.

**Methods:**

In total, 117,054 individuals from the Copenhagen City Heart Study and the Copenhagen General Population Study (the Copenhagen studies) and 390,374 individuals from UK Biobank were included for observational and one-sample Mendelian randomisation (MR) analyses. Fragility fractures were defined as fractures at the hip, spine and arm (humerus/wrist), collected from national health registries. Summary data for fasting glucose and HbA_1c_ concentrations from 196,743 individuals in the Meta-Analyses of Glucose and Insulin-related traits Consortium (MAGIC) were combined with data on fragility fractures from the Copenhagen studies in two-sample MR analyses.

**Results:**

Higher fasting and non-fasting glucose and HbA_1c_ concentrations were associated with higher risk of any fragility fracture (*p*<0.001). Individuals with vs without diabetes had HRs for fragility fracture of 1.50 (95% CI 1.19, 1.88) in type 1 diabetes and 1.22 (1.13, 1.32) in type 2 diabetes. One-sample MR supported a causal association between high non-fasting glucose concentrations and increased risk of arm fracture in the Copenhagen studies and UK Biobank combined (RR 1.41 [1.11, 1.79], *p*=0.004), with similar results for fasting glucose and HbA_1c_ in two-sample MR analyses (ORs 1.50 [1.03, 2.18], *p*=0.03; and 2.79 [1.12, 6.93], *p*=0.03, respectively). The corresponding MR estimates for any fragility fracture were 1.18 (1.00, 1.41), *p*=0.06; 1.36 (0.89, 2.09), *p*=0.15; and 2.47 (0.95, 6.43), *p*=0.06, respectively. At age 80 years, cumulative death was 27% in individuals with fragility fracture only, 54% in those with diabetes only, 67% in individuals with both conditions and 17% in those with neither.

**Conclusions/interpretation:**

Hyperglycaemia and diabetes are risk factors for fragility fracture and one- and two-sample MR analyses supported a causal effect of hyperglycaemia on arm fractures. Diabetes and previous fragility fracture jointly conferred the highest risk of death in the general population.

**Graphical Abstract:**

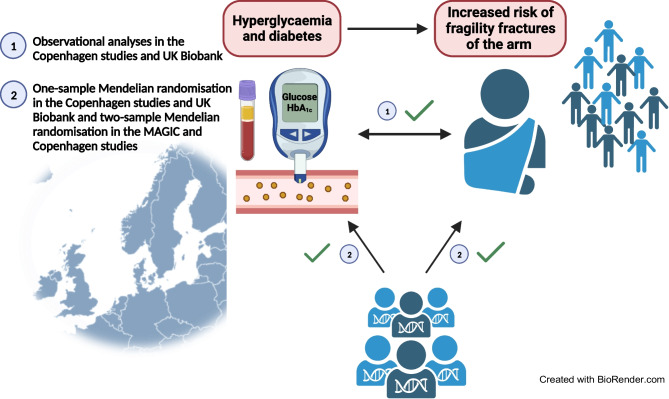

**Supplementary Information:**

The online version of this article (10.1007/s00125-023-06054-8) contains peer-reviewed but unedited supplementary material.


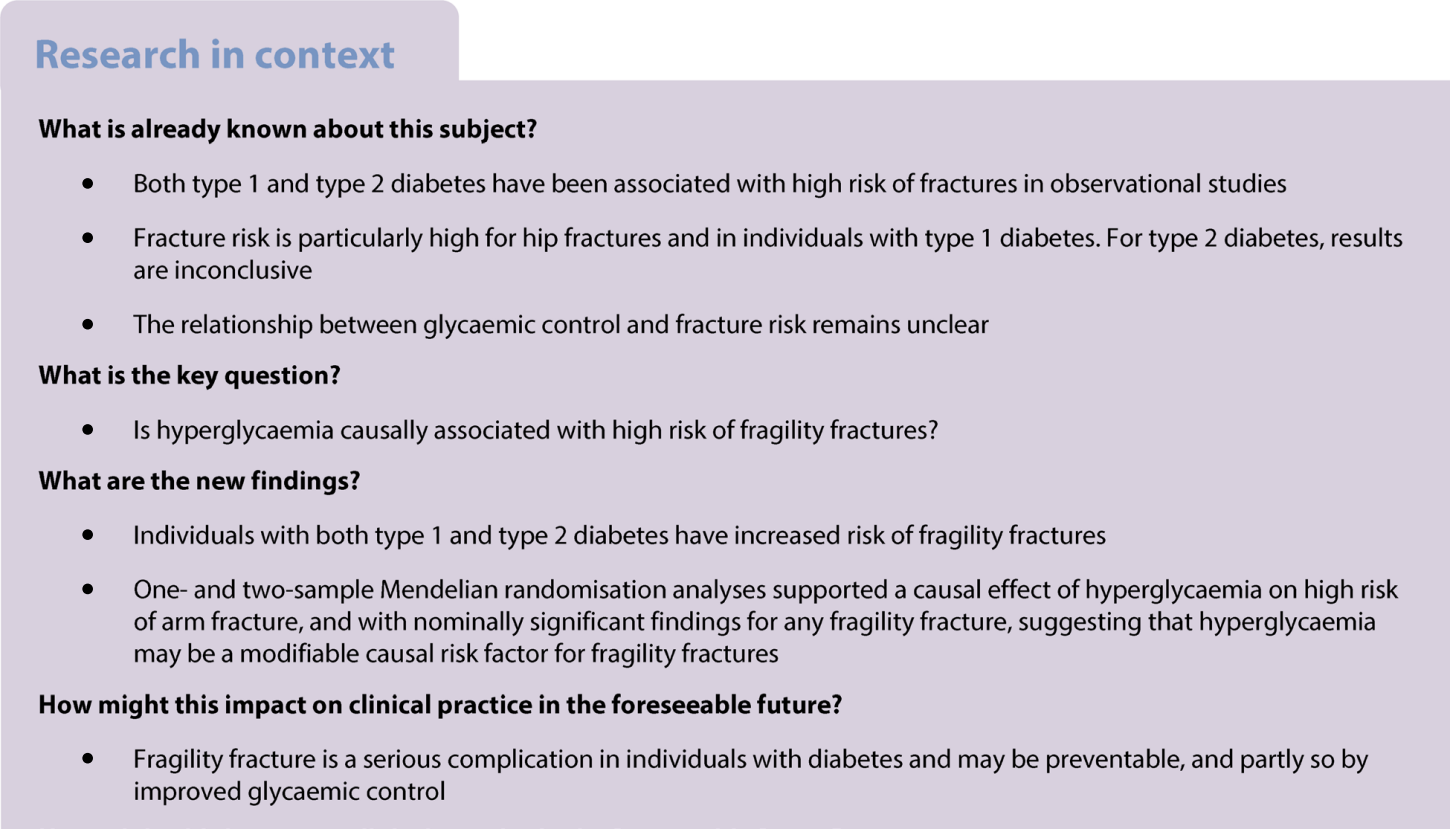


## Introduction

Diabetes is a major contributor to morbidity and mortality globally. Similarly, fragility fractures result in prolonged immobilisation and hospitalisation, and have a significant impact on morbidity, mortality and quality of life. Presently, and increasingly with the growth of the ageing population, both diabetes and fragility fractures constitute a substantial healthcare burden and may be linked. Several observational studies indicate that individuals with type 1 and type 2 diabetes may have increased risk of fractures [1, 2]. In type 1 diabetes, the higher risk could be attributable to relative lack of insulin and its anabolic effects on the skeleton, leading to a lower peak bone mass [3]. Individuals with type 2 diabetes often have normal or high bone mineral density but a reduced bone turnover, which impairs bone quality and may partly explain a higher risk of fractures [4]. A pathophysiological mechanism leading to high fragility fracture risk could involve harmful effects of chronic hyperglycaemia on the microvasculature, as is well known from eye, nerve and kidney complications in diabetes, affecting bone metabolism and bone vasculature per se [5, 6]. However, shared risk factors between diabetes and fragility fractures, such as old age, medication use, falls due to hypoglycaemia, poor eyesight and peripheral neuropathy, or side effects of glucose-lowering drugs such as thiazolidinediones may confound this association [3].

Mendelian randomisation (MR) is an epidemiological approach that uses genetic variants strongly associated with a specific phenotype to compare the risk of an outcome in population subgroups stratified by the genotype. Due to the independent segregation of alleles from parents to offspring at conception, the population subgroups by genotype will differ systematically only in terms of the specific phenotype, and confounding factors will be largely evenly distributed, comparable to a randomised controlled trial [7]. Thus, MR provides estimates largely free of confounding and reverse causation and is a valuable complement in the assessment of causal relationships between a phenotype and a disease, in addition to conventional observational analyses, which may be confounded and influenced by reverse causation, and randomised controlled trials, which may be difficult, expensive and sometimes unethical to perform [8].

In a large general population setting, using both observational and one- and two-sample and bidirectional MR designs, we tested the hypotheses that: (1) individuals with hyperglycaemia and diabetes have increased risk of fragility fracture; (2) hyperglycaemia is causally associated with increased risk of fragility fracture; and (3) diabetes and fragility fracture jointly associate with the highest risk of death.

## Methods

### Study design and participants

We included 117,054 individuals from two Copenhagen studies, the Copenhagen City Heart Study (CCHS) and the Copenhagen General Population Study (CGPS), for observational and one- and two-sample MR analyses; up to 390,374 individuals from UK Biobank for observational and one-sample MR analyses; and summary data from up to 196,734 individuals in the Meta-Analyses of Glucose and Insulin-related traits Consortium (MAGIC) for two-sample MR analyses.

### CCHS and CGPS: the Copenhagen studies

The CCHS was initiated in 1976–1978 with follow-up examinations in 1981–1983, 1991–1994, 2001–2003 and 2011–2014. Participants were selected based on the national Danish Civil Registration System to reflect the Danish adult population aged 20–100 years. Data were obtained from a self-administrated questionnaire (including self-identified gender), which was reviewed together with an investigator at the day of attendance; from a physical examination; and from blood samples including DNA extraction [9]. In the present study, 10,696 individuals from the 1991–1994 and 2001–2003 examinations of the CCHS were included.

The CGPS was initiated in 2003 and enrolment is ongoing. Participants were selected and examined as described for the CCHS. In total, 106,358 individuals from the CGPS were included in the present study.

All participants in the Copenhagen studies were white and of Danish descent (self-reported), and none were included in more than one study. Both studies were approved by institutional boards and Danish ethical committees (KF-100.2039/91, KF-01-144/01, H-KF-01-144/01) and were conducted according to the Declaration of Helsinki. Written informed consent was obtained from all individuals.

### UK Biobank

UK Biobank was initiated in 2006 and is a prospective general population study including approximately 500,000 individuals between 40 and 69 years of age from the UK. Data collection at the initial assessment visit included a self-administrated questionnaire (including self-identified gender), physical measurements and blood samples including DNA extraction [10]. Written informed consent was obtained from all individuals along with ethics approval from the North West Multicentre Research Ethics Committee (MREC). In total, 390,374 individuals of white British descent with an available HbA_1c_ measurement were included for observational analyses and 359,159 individuals with an available glucose measurement were included for one-sample MR analyses.

### MAGIC

Publicly available summary-level data on fasting glucose and HbA_1c_ concentrations from up to 196,734 individuals without diabetes and of European descent were collected from MAGIC. Neither the Copenhagen studies nor UK Biobank were part of MAGIC [11].

### Plasma glucose, HbA_1c_ and covariates

Plasma glucose was measured by colorimetric assays (Konelab, Boehringer Mannheim, Germany) in the Copenhagen studies and by hexokinase analysis (Beckman Coulter, Brea, CA, USA) in UK Biobank. HbA_1c_ in UK Biobank was measured by high-performance liquid chromatography (VARIANT II Turbo, Bio-Rad, Hercules, CA, USA). In the Copenhagen studies, 116,076 individuals had an available glucose measurement (0.8% missing). In UK Biobank, 359,159 of 409,554 individuals had an available glucose measurement at the initial or first repeat assessment visit (12% missing) and 390,374 had an available HbA_1c_ measurement (4.7% missing). For all individuals, blood samples were taken at random irrespective of time since the last meal. BMI was calculated as weight in kilograms divided by measured height in metres squared. Information on current smoking, pack-years smoked, alcohol intake, physical activity, education, time since last meal in the Copenhagen studies and menopausal status for women was self-reported. Type 1 and type 2 diabetes were defined according to the World Health Organization’s ICD codes, eighth and tenth revisions (ICD-8, ICD-10 [http://apps.who.int/classifications/icd10/browse/2016/en]) for the Copenhagen studies and ninth and tenth revisions (ICD-9 [http://www.icd9data.com/2007/Volume1/default.htm], ICD-10) for UK Biobank, and codes were collected from national health registries.

### Genotypes

Seven genetic variants with well-established associations with high glucose and HbA_1c_ concentrations in genome-wide association studies were selected as genetic instruments [11–13]. The variants were selected as they represent independent loci showing strong associations between the genotype and plasma glucose and in genes known to be involved in glucose metabolism: *G6PC2* (rs560887), *GCK* (rs4607517), *DGKB* (rs2191349), *ADCY5* (rs11708067), *CDKN2A* and CDKN2B (rs1081166 and rs2383206) and *TCF7L2* (rs7903146) (electronic supplementary material [ESM] Table 1). A targeted selection approach for genetic variants was chosen to reduce the risk of pleiotropy [7, 14]*.* An ABI PRISM 7900HT Sequence Detection System and TaqMan-based assays (both Applied Biosystems, Foster City, CA, USA) were used to genotype the variants in the Copenhagen studies. Large-scale sequencing (genotype arrays) in all individuals has not been performed in the Copenhagen studies. In UK Biobank, six of the seven variants were available (rs2383206 was not genotyped) and had been genotyped on two closely related purpose-designed arrays, the UK BiLEVE Axiom array and the UK Biobank Axiom array (Thermo Fisher Scientific, Applied Biosystems, Foster City, CA, USA). A proxy of rs2383206, rs2383207 (*r*^2^=0.99, *D*ʹ=1.00 with rs2383206), was identified by the web-based tool SNiPA (http://snipa.org/snipa3/; accessed 30 October 2023) and was used instead of rs2383206 in UK Biobank. Neither the Copenhagen studies nor UK Biobank were part of the genome-wide association studies in which the variants were discovered [11, 13]. All genotype data were quality controlled and tested for Hardy–Weinberg equilibrium. For individuals with available genotypes at the seven loci (113,540 individuals in the Copenhagen studies and 359,159 individuals in UK Biobank) weighted allele scores were constructed by coding genotypes 0, 1 and 2 for non-carriers, heterozygous carriers and homozygous carriers of the glucose-increasing alleles, respectively, and multiplying with the percentage higher glucose for each allele compared with the reference non-carrier allele for each individual variant, as done previously [15, 16]. The weighted allele scores were used for one-sample MR analyses in the Copenhagen studies and UK Biobank, respectively. For the two-sample MR analyses, β-coefficients for the associations with fasting plasma glucose and HbA_1c_ for the seven genetic glucose variants were collected from MAGIC [11] and β-coefficients for the associations with any fracture, hip fracture and arm fracture were estimated in the Copenhagen studies. For two-sample, bidirectional MR analyses, investigating a potential causal effect of fracture risk on plasma glucose, HbA_1c_ and risk of type 1 and type 2 diabetes, we used 14 genetic variants associated with risk of fracture at genome-wide significance level in UK Biobank, and replicated in the 23andMe cohorts [17]. β-Coefficients for the association with fracture risk for these 14 variants were collected and harmonised with publicly available β-coefficients for the associations with fasting plasma glucose, 2 h post challenge plasma glucose and HbA_1c_ in MAGIC [18]; for the association with type 2 diabetes from the Diabetes Meta-Analysis of Trans-Ethnic association studies (DIAMANTE) consortium [19]; and for type 1 diabetes from a genome-wide association study by Forgetta et al [20]. All analyses were restricted to individuals of European descent (as stated by the investigators).

### Outcomes

Fragility fractures were defined as ICD diagnosis codes for fractures of the hip, spine and arm (proximal humerus and wrist). For detailed ICD codes, see ESM Table 2. The endpoint ‘any fragility fracture’ represents a composite of the three fracture types, and an individual was defined as having a fragility fracture at the first event of any fracture type (hip, spine or arm). Due to relatively low numbers of spine fractures (*n*=905), analyses for spine fractures were not performed separately but only included in the composite endpoint of any fragility fracture. In the Copenhagen studies, ICD-8 and ICD-10 codes for fragility fracture and all-cause death were collected from the national Danish Patient Registry and the national Danish Central Person Registry, from 1 January 1977 to 13 December 2018. ICD-9 was not used as this revision of the ICD was never introduced in Denmark, and ICD-10 directly replaced ICD-8 as of 1 January 1994. In UK Biobank, fragility fractures were defined by the corresponding ICD-9 and ICD-10 diagnosis codes collected from the Hospital Inpatient Data field, which links to inpatient hospital data registries from England, Wales and Scotland (https://biobank.ctsu.ox.ac.uk/crystal/ukb/docs/inpatient_mapping.pdf). In observational analyses, follow-up began at the first inclusion into the study and ended with censoring at the date of death, event or emigration, or on 13 December 2018 in the Copenhagen studies (corresponding to the end of follow-up for the least updated register) and on 30 September 2021 in UK Biobank (corresponding to the latest update of the Hospital Inpatient Data field). In the Copenhagen studies, during a median of 10 years of follow-up (range: 0.1–43), 8466 individuals had a fragility fracture (only first event counted), 905 a spine fracture, 2746 a hip fracture and 6187 an arm fracture. In UK Biobank, during a median follow-up time of 14 years (range: 0–16), 4534 individuals had a fragility fracture (only first event included), 1868 a spine fracture, 1389 a hip fracture and 2696 an arm fracture. As an individual’s genotype is constant throughout life, the MR analyses included all registered fragility fractures, i.e. including events registered before the date the individual entered the study (Copenhagen studies or UK Biobank) in individuals with available genotypes. In the Copenhagen studies this yielded 15,349 individuals with any fragility fracture, 1378 with spine fractures, 3474 with hip fractures and 12,076 individuals with arm fractures; in UK Biobank, corresponding numbers were 13,971, 2055, 4074 and 8626 individuals, respectively.

### Statistical analyses

We used Stata SE version 17.0 (StataCorp, College Station, TX, USA) for all statistical analyses. Deviation from Hardy–Weinberg equilibrium was tested using Pearson’s χ^2^ test. As our analyses included three endpoints, a multiple-testing-corrected (by the Bonferroni method, 0.05/3) *p* value of *p*<0.02 was considered statistically significant.

The observational associations between continuous non-fasting and fasting glucose (Copenhagen studies) and HbA_1c_ concentrations (UK Biobank) and risk of any fragility fracture, hip fracture, arm fracture and death were examined using restricted cubic splines with five knots incorporated in a Cox proportional hazards model. The population median concentrations of 5.2 mmol/l for glucose and 35.2 mmol/mol (5.4%) for HbA_1c_ were used as references. To test whether a diagnosis of type 1 or type 2 diabetes was associated with increased risk of any fragility fracture, hip fracture or arm fracture in the Copenhagen studies, we used Cox proportional hazards models with age as the time scale and with delayed entry at examination (left truncation). The models were adjusted for sex, birth year (to accommodate for time difference in initiation between the CCHS and the CGPS), BMI, current smoking, physical activity, alcohol intake, time since last meal in the Copenhagen studies and menopausal status for women. In the Copenhagen studies, missing values for covariates (0–2.4% missing) were imputed from age, sex and cohort (CCHS or CGPS) using multivariate imputation (Stata’s *mi impute* command); missing values (0–27%) were not imputed in UK Biobank.

The associations of the genetic variants and weighted allele scores with glucose concentrations were tested using linear regression. To test for trend across the weighted allele scores in quintiles and non-fasting glucose, fasting glucose and HbA_1c_, we used the non-parametric Cuzick’s extension of a Wilcoxon rank sum test. One-sample MR analyses of the potential causal effect of glucose concentration on any fragility fracture, hip fracture and arm fracture risk were performed using instrumental variable analysis and two-stage least-squares regression by the publicly available *ivreg2* and *ivpois* commands in Stata [21, 22]. The strength of the genetic instrument (i.e. the association between weighted allele scores and glucose concentrations) was confirmed by *F* statistics in the first-stage regression [8, 23]. Potential pleiotropic effects of the weighted allele score on fragility fracture risk through the measured confounders BMI, alcohol intake, smoking and level of physical activity were investigated using linear regression, where a significant association between the genotype and confounder could indicate pleiotropy. A combined causal MR estimate for the Copenhagen studies and UK Biobank was estimated by meta-analyses using the *metan* command. Between-study heterogeneity was assessed by *I*^2^ statistics. A fixed effects model was chosen as between-study heterogeneity was low to moderate (*I*^2^=0–43%) [24]. Two-sample bidirectional MR analyses were performed: (1) for the same seven genetic glucose variants and fracture endpoints; and (2) for the 14 genetic fracture variants and fasting plasma glucose, 2 h post challenge plasma glucose, HbA_1c_ and type 1 and type 2 diabetes using inverse variance-weighted, MR Egger and MR median regression. Finally, mortality percentages as a function of age were plotted using a Kaplan–Meier estimator approach, and differences across ordered categories of diabetes and fragility fracture status, and by glucose categories, were examined using the logrank test.

## Results

In total, 117,054 individuals from the Copenhagen studies and 390,374 individuals from UK Biobank were included for observational and one-sample MR analyses. Baseline characteristics for individuals by study and diabetes status in the Copenhagen studies are shown in Table 1. In the Copenhagen studies, individuals with vs without diabetes were older, had higher BMI and glucose concentrations and had smoked more pack-years. A larger fraction of individuals with type 1 and type 2 diabetes had low physical activity level, and a smaller fraction had moderate physical activity level, compared with individuals without diabetes (Table 1). Individuals in the Copenhagen studies and UK Biobank were of similar age at study entry, and with similar percentages of men and women. Individuals in the Copenhagen studies had lower BMI, but higher glucose concentrations, compared with individuals in UK Biobank. Individuals in the Copenhagen studies were more often smokers and had lower levels of physical activity, compared with individuals in UK Biobank. Individuals in UK Biobank more often had diabetes, but fewer individuals had a fragility fracture, compared with individuals in the Copenhagen studies. Genotype distributions did not deviate from Hardy–Weinberg expectations (all *p*>0.05).

**Table 1:** Baseline characteristics for individuals in the CCHS and the CGPS (Copenhagen studies) and UK Biobank

Characteristic	Copenhagen studies				UK Biobank
	All	No diabetes	Type 1 diabetes	Type 2 diabetes	All
Number of individuals	117,054	110,028 (94)	594 (0.5)	6432 (5)	390,374
Age (years)	57 (47–67)	56 (47–66)	58 (49–70)	63 (54–71)	58 (51–63)
Sex (women)	64,119 (55)	61,042 (55)	308 (52)	2769 (43)	210,967 (54)
BMI (kg/m^2^)	25.6 (23.2–28.4)	25.4 (23.1–28.1)	25.7 (23.6–28.9)	29.1 (26.3–32.7)	26.7 (24.2–29.9)
Glucose (mmol/l)	5.2 (4.7–5.7)	5.1 (4.7–5.7)	5.9 (5.0–8.3)	6.3 (5.4–7.8)	4.9 (4.6–5.3)
HbA_1c_ (mmol/mol)					35.2 (32.7–37.8)
HbA_1c_ (%)					5.4 (5.1–5.6)
Alcohol (units/week)	8 (3–15)	8 (3–15)	8 (3–15)	7 (2–16)	9 (5–15)
Pack-years (smokers)	15 (5–30)	14 (5–29)	19 (8–35)	23 (10–40)	20 (10–33)
Physical activity					
Low	34,727 (30)	31,756 (29)	211 (36)	2760 (43)	92,107 (32)
Moderate	79,169 (68)	75,325 (68)	364 (61)	3480 (54)	148,294 (53)
High	2782 (2)	2641 (2)	16 (3)	125 (2)	43,862 (15)
Type 1 diabetes	594 (0.5)	–	–	–	3618 (0.9)
Type 2 diabetes	6432 (5)	–	–	–	30,433 (8)
Fragility fracture, any	15,815 (14)	14,520 (13)	123 (21)	1172 (18)	13,971 (4)
Hip fracture	3587 (3)	3150 (3)	52 (9)	385 (6)	4074 (1)
Arm fracture	12,435 (11)	11,516 (10)	80 (13)	839 (13)	8626 (2)

Data are absolute numbers (%) for categorical variables and median (IQR) for continuous variables

The Copenhagen studies baseline characteristics are shown for all individuals and by diabetes status. The UK Biobank baseline characteristics are shown for all individuals. Glucose measurements were performed on non-fasting samples. Units of alcohol/week is for individuals currently drinking alcohol. Pack-years are for current and former smokers. Self-reported physical activity was available in 284,263 individuals in UK Biobank. Diagnoses of type 1 diabetes, type 2 diabetes and fragility fractures were defined by ICD-8, ICD-9 and ICD-10 diagnosis codes and were collected from national registries

### Glucose concentrations and fragility fracture: observational analyses

**Fig. 1:**
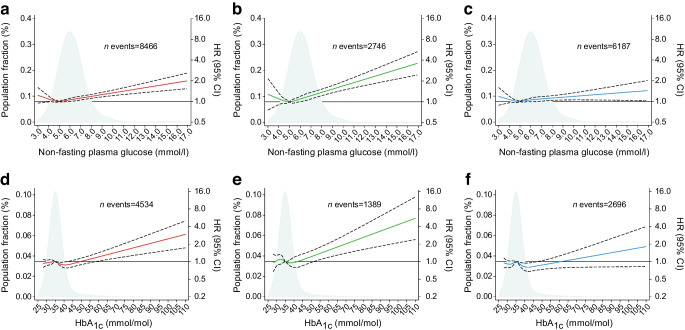
Risk of any fragility fracture (**a**, **d**), hip fracture (**b**, **e**) and arm fracture (proximal humerus and wrist) (**c**, **f**) as a function of non-fasting glucose (**a**–**c**) and HbA_1c_ concentrations (**d**–**f**) in the Copenhagen studies and in UK Biobank, respectively, by restricted cubic spline analyses incorporated into a Cox proportional hazards model and adjusted for sex, birth year, BMI, current smoking, physical activity level, units of alcohol drunk per week, time since last meal for non-fasting glucose and menopausal status for women**.** Solid red, green and blue lines denote HRs and broken lines 95% CIs. The solid black line denotes HR=1 for comparison. The reference was set to the population median (5.2 mmol/l for glucose and 35.2 mmol/mol [5.4%] for HbA_1c_). The light-blue area shows the distribution of glucose and HbA_1c_ concentrations in the population

Figure 1 shows the prospective risk of any fragility fracture (Fig. 1a,d), hip fracture (Fig. 1b,e) and arm fracture (Fig. 1c,f) as a function of glucose concentration at study entry in the Copenhagen studies (Fig. 1a–c) and as a function of HbA_1c_ concentration at study entry in UK Biobank (Fig. 1d–f) by restricted cubic spline analyses. Compared with the population median glucose concentration of 5.2 mmol/l in the Copenhagen studies, higher glucose concentrations were associated with higher risk of any fragility fracture, hip fracture and arm fracture (*p* for all <0.001), also below the cut-off for diabetes (random plasma glucose <11.1 mmol/l) (Fig. 1a–c). The corresponding analyses including only individuals with fasting glucose concentrations showed similar results (ESM Fig. 1). Compared with the population median for HbA_1c_ of 35.2 mmol/mol (5.4%) in UK Biobank, higher HbA_1c_ concentrations were associated with higher risk of any fragility fracture and hip fracture (*p* for both <0.001), but not with higher risk of arm fracture (*p*=0.32) (Fig. 1d–f).

### Diabetes and fragility fracture: observational analyses

**Fig. 2:**
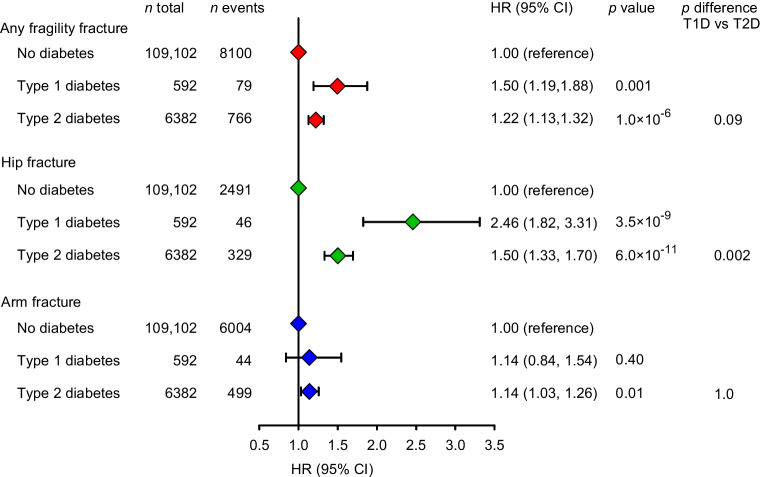
Risk of any fragility fracture, hip fracture and arm fracture (proximal humerus and wrist) in the Copenhagen studies according to diabetes. Estimates were derived by a Cox proportional hazards model, adjusted for sex, birth year, BMI, current smoking, physical activity level, units of alcohol per week, time since last meal and menopausal status for women. T1D, type 1 diabetes; T2D, type 2 diabetes

Figure 2 shows the risk of any fragility fracture, hip fracture and arm fracture in the Copenhagen studies as a function of diabetes status. Individuals with type 1 and type 2 diabetes had higher risk of any fragility fracture compared with individuals without diabetes, with HRs of 1.50 (95% CI 1.19, 1.88) and 1.22 (1.13, 1.32), respectively (Fig. 2). Corresponding estimates for hip fracture were 2.46 (1.82, 3.31) and 1.50 (1.33, 1.79) and for arm fracture 1.14 (1.03, 1.26) and 1.14 (0.84, 1.54), respectively.

### Glucose concentrations and fragility fracture: one-sample, two-sample and bidirectional MR analyses

**Fig. 3:**
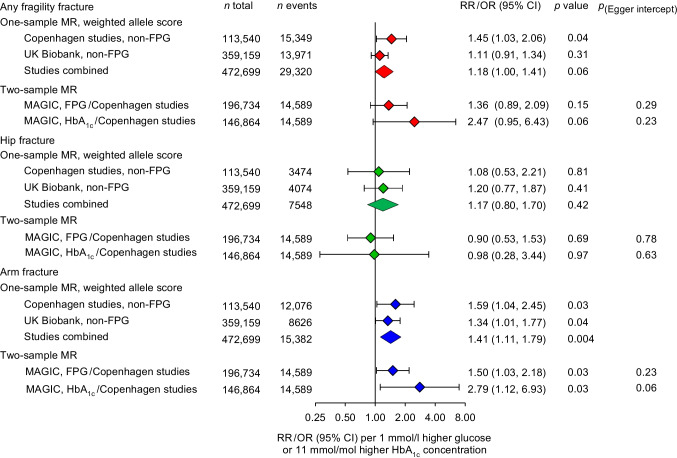
One-sample MR analysis of risk of any fragility fracture, hip fracture and arm fracture (proximal humerus and wrist) in the Copenhagen studies, in UK Biobank and in the studies combined for a 1 mmol/l higher non-fasting plasma glucose (non-FPG) concentration. Estimates were derived by instrumental variable analysis based on a weighted allele score of seven glucose genetic variants and adjusted for age and sex. Two-sample MR analysis of risk of any fragility fracture, hip fracture and arm fracture (proximal humerus and wrist) was performed with β-coefficients for fasting plasma glucose (FPG) and HbA_1c_ from MAGIC and β-coefficients for fractures from the Copenhagen studies. Estimates were derived by inverse variance-weighted MR for the same seven variants as used in the one-sample MR analyses. The *p* value for the Egger intercept was derived from MR Egger regression

The selected genetic variants in *G6PC2*, *GCK*, *DGKB*, *ADCY5*, *CDKN2A/ CDKN2B* and *TCF7L2* were, separately and combined into a weighted allele score, associated with higher glucose concentrations in the Copenhagen studies and UK Biobank in a concordant manner (ESM Table 3). Instrument strengths for the weighted allele scores were confirmed by *F* values of 558 in the Copenhagen studies and 2081 in UK Biobank [8]. The weighted allele scores explained 0.4% and 0.6% of the variation in plasma glucose in the Copenhagen studies and UK Biobank, respectively. The weighted allele score was associated with non-fasting glucose, fasting glucose, HbA_1c_ and higher risk of type 2 diabetes in a similar manner in the Copenhagen studies and in UK Biobank (ESM Fig. 2). In one-sample MR analyses, the RR of any fragility fracture per 1 mmol/l higher glucose concentration was 1.45 (95% CI 1.03, 2.06), *p*=0.04, in the Copenhagen studies; 1.11 (0.91, 1.34), *p*=0.31, in UK Biobank; and 1.18 (1.00, 1.41), *p*=0.06, in meta-analysis of the combined studies (Fig. 3). For hip fracture the corresponding estimates were 1.08 (0.53, 2.21), *p*=0.81; 1.20 (0.77, 1.87), *p*=0.41; and 1.17 (0.80, 1.70), *p*=0.42; and for arm fracture 1.59 (1.04, 2.45), *p*=0.03; 1.34 (1.01, 1.77), *p*=0.04; and 1.41 (1.11, 1.79), *p*=0.004, respectively.

The associations between the weighted allele scores and the potential confounders alcohol, smoking and physical activity showed no indication of pleiotropy, with similar results for both studies (ESM Table 4). There was an association between the weighted allele score and BMI in both studies (ESM Table 4), suggestive of a potential pleiotropic effect (*p*=0.002 in the Copenhagen studies and *p*=0.0001 in UK Biobank) (ESM Table 4). We therefore performed an additional MR analysis adjusted for BMI, which showed similar results as the main analyses (compare Fig. 3 and ESM Fig. 3).

In two-sample MR inverse variance-weighted analyses, the OR of any fragility fracture per 1 mmol/l higher fasting glucose concentration was 1.36 (0.89, 2.09), *p*=0.15; for hip fracture 0.90 (0.53, 1.53), *p*=0.69; and for arm fracture 1.50 (1.03, 2.18), *p*=0.03 (Fig. 3). The corresponding estimates per 11 mmol/mol (1.0%) higher HbA_1c_ were 2.47 (0.95, 6.43), *p*=0.06; 0.98 (0.28, 3.44), *p*=0.97; and 2.79 (1.12, 6.93), *p*=0.03, respectively. MR Egger and weighted median regression showed similar results with no indication of pleiotropy or bias due to invalid instruments (ESM Table 5). Genetically predicted fracture risk did not associate with fasting plasma glucose, 2 h post challenge plasma glucose, HbA_1c_ or with type 1 or type 2 diabetes (ESM Fig. 4).

### Diabetes, fragility fracture and all-cause mortality: observational analyses

**Fig. 4:**
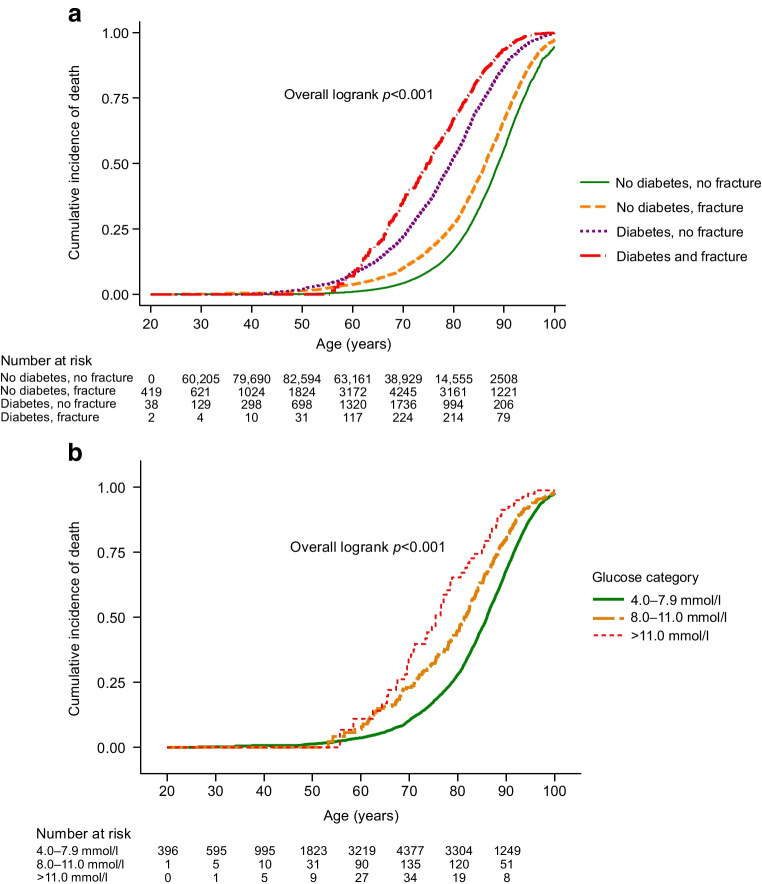
Cumulative incidence of death by age and diabetes/fragility fracture status in all individuals (**a**) and by glucose in categories in individuals with previous fragility fracture (**b**) in the Copenhagen studies

The cumulative incidence of death as a function of age was stepwise higher in individuals with previous fragility fracture, with diabetes and with diabetes and previous fragility fracture combined, compared with individuals without diabetes or fragility fracture (overall logrank *p*<0.001) (Fig. 4a). At age 80 years, cumulative death was 17% in individuals with neither diabetes nor fragility fracture, 27% in individuals with previous fragility fracture and no diabetes, 54% in individuals with diabetes and no previous fragility fracture and 67% in individuals with both diabetes and previous fragility fracture.

In individuals with a previous fragility fracture, cumulative incidence of death as a function of age was higher with higher glucose concentrations (overall logrank *p*<0.001) (Fig. 4b). Similarly, compared with the population median glucose concentration of 5.2 mmol/l, higher glucose concentrations were associated with higher risk of death (ESM Fig. 5). At age 80 years, the cumulative incidence of death was 28% in individuals with glucose of 4.0–7.9 mmol/l, 45% in individuals with glucose of 8.0–11.0 mmol/l and 67% in individuals with glucose above 11.0 mmol/l (overall logrank *p*<0.001) (Fig. 4b).

## Discussion

In observational analyses of 117,054 individuals in the Danish general population and in 390,374 individuals in the British general population, we found that higher glucose and HbA_1c_ concentrations were associated with higher risk of fragility fracture and that individuals with both type 1 and type 2 diabetes had a higher risk of fragility fracture compared with individuals without diabetes. One- and two-sample MR analyses supported a causal association between high glucose and HbA_1c_ concentrations and risk of arm fracture. Finally, diabetes and previous fragility fracture jointly conferred the highest all-cause mortality in the general population. The totality of our results represents novel findings and insight.

Mechanistically, the present findings may be explained by a harmful effect of hyperglycaemia on bone microvasculature and bone turnover, potentially through damage to the vascular endothelium and glycation of collagens, leading to the accumulation of advanced glycation end-products and impaired bone quality [5, 25].

Several studies and meta-analyses have reported higher fracture risk at different skeletal sites in individuals with diabetes, with up to eightfold higher risk of hip fracture in individuals with type 1 diabetes and up to 1.4-fold higher risk in individuals with type 2 diabetes [1, 2]. However, a recent large study from the UK found no difference in risk of any fracture in individuals with type 2 diabetes compared with individuals without [26]. In the present study, we chose to investigate fractures of the hip, spine and arm (proximal humerus and wrist) as they represent common sites for low-energy, fragility fractures.

Novel findings of the present study include that we observed a linear relationship between higher glucose concentrations above approximately 6–7 mmol/l and increased risk of fragility fracture, and that fragility fracture and diabetes combined conferred the highest mortality in individuals in the general population. In line with previous studies, we found that individuals with type 1 and type 2 diabetes have higher risk of fragility fracture compared with individuals without; however, our risk estimates are modest, especially for type 1 diabetes, compared with those of previous smaller, cross-sectional studies [27]. Further, another novel observation in the present study in the MR analysis was that we found support of a causal association between high glucose concentrations and increased risk of arm fracture.

Why there may be a causal impact of hyperglycaemia on risk of arm fracture and not on hip fracture is not clear and warrants further studies. One difference is that bones of the hip are weight-bearing, whereas those of the arms are not. According to this difference, hyperglycaemia has been shown to inhibit osteogenesis, and bone formation markers have been shown to be inversely associated with HbA_1c_ and fasting plasma glucose in individuals with and without diabetes [3, 6, 28]. Further, the inhibition of osteogenesis is counterbalanced by mechanical stimulation [3], which is more regular in weight-bearing bones compared with non-weight-bearing bones. A second difference is that there may also be anatomical distinctions in terms of vascular supply and microvascular impairment, a difference that may affect the bone of arms and hip differently.

A potential limitation to our study is that glucose measurements were performed on plasma samples obtained in the non-fasted state; however, it can also be argued that this is a strength as the non-fasting state is present in more hours of a 24 h cycle than the fasting state [29]. Thus, we could not classify individuals according to impaired fasting glucose or impaired glucose tolerance status. However, observational analyses limited to individuals with fasting glucose concentrations in the Copenhagen studies showed similar results. Also, the two-sample MR analyses with fasting glucose summary data from the MAGIC consortium showed similar results as in the one-sample MR based on non-fasting glucose. In the MR analyses, we had 80% power to detect an OR of 1.4 or higher for risk of fragility fracture per 1 mmol/l higher plasma glucose [30]. Thus, our study is underpowered to detect smaller causal effects. A potential limitation of the MR analyses is that the included genetic variants may have pleiotropic effects, which might affect the studied outcome (i.e. fragility fracture risk) through other, glucose-independent, pathways. In our selection of genotypes, we sought to select genetic variants with well-known effects on beta cell function and insulin secretion, and we found no indication of pleiotropy from the associations between the weighted allele score and smoking, alcohol intake or level of physical activity. In the two-sample MR analysis, we found no indication of pleiotropy in the MR Egger regression. However, as glucose concentrations are causally associated with higher risk of peripheral neuropathy, we cannot exclude that the higher risk of fractures observed in the MR analyses is partly due to increased risk of falls due to peripheral neuropathy or poor balance, and not solely due to a direct effect of hyperglycaemia on the bone. Hypoglycaemic episodes in individuals with diabetes may also increase the risk of falls and fractures and thus bias the results. In the MR analyses, this would, however, only result in vertical pleiotropy, as peripheral neuropathy, hypoglycaemia and poor balance are phenotypic changes on the same pathway coming from hyperglycaemia (i.e. hierarchically related events). Also, our study is limited to individuals of European descent and may not be generalisable to other populations.

Strengths of our study include: (1) the large number of phenotypically well-characterised and genetically homogenous individuals included in both prospective observational and one- and two-sample MR study designs; and (2) access to highly valid, long-term follow-up data from nationwide registries without losses to follow-up.

In conclusion, hyperglycaemia and diabetes are risk factors for fragility fracture, and MR analyses supported a causal effect of high glucose concentrations on fragility fracture of the arm. Diabetes and previous fragility fracture jointly conferred the highest all-cause mortality in the general population. The results suggest that hyperglycaemia may be a modifiable causal risk factor of fragility fracture. However, whether improved glycaemic control lowers fragility fracture risk needs to be tested in randomised controlled trials.

### Supplementary Information

Below is the link to the electronic supplementary material.

Supplementary file1 (PDF 1308 KB)

## Data Availability

Aggregated data from the Copenhagen City Heart Study and the Copenhagen General Population Study may be available on reasonable request to the corresponding author. UK Biobank is a publicly available resource, available upon application to UK Biobank. Summary data from the Meta-Analyses of Glucose and Insulin-related traits Consortium are publicly available.

## Abbreviations

CCHS: Copenhagen City Heart Study
CGPS: Copenhagen General Population Study
MAGIC: Meta-Analyses of Glucose and Insulin-related traits Consortium
MR: Mendelian randomisation
