# Supplementary material for: Hyperglycaemia, diabetes and risk of fragility fractures: observational and Mendelian randomisation studies
Source: Diabetologia. 2023 Dec 14;67(2):301–11. doi: 10.1007/s00125-023-06054-8 (PMC10789835; doi:10.1007/s00125-023-06054-8)
Supplement: Supplementary file 1 — Supplementary file1 (PDF 1308 KB) [file 125_2023_6054_MOESM1_ESM.pdf]

Electronic supplementary material (ESM)

Hyperglycaemia, diabetes and risk of fragility fractures: observational and Mendelian randomisation studies

Frida Emanuelsson[1,2], Shoaib Afzal[2,3,4], Niklas R. Jørgensen[1,2], Børge G. Nordestgaard[2,3,4], Marianne Benn[1,2,4]

[1]Department of Clinical Biochemistry, Copenhagen University Hospital Rigshospitalet, Centre of Diagnostic Investigation, Copenhagen, and Glostrup, Denmark

[2] Department of Clinical Medicine, Faculty of Health and Medical Sciences, University of Copenhagen, Copenhagen, Denmark

[3]Department of Clinical Biochemistry, Copenhagen University Hospital Herlev and Gentofte, Herlev, Denmark

[4]The Copenhagen General Population Study, Copenhagen University Hospital Herlev and Gentofte, Herlev, Denmark.

Supplementary Table 1.

Overview of genes, their involvement in glucose metabolism and associated tag single nucleotide polymorphisms (SNPs) used as instrument for glycaemia in the current study.

Gene	Genomic coordinates (GRCh38)	Encoded gene product	Function/involvement in glucose metabolism	References for function	Tag SNP (Rsid)	Tag SNP position (GRCh38)	Type of variant
G6PC2	2:168901291-168910000	Islet-specific glucose-6-phosphatase catalytic subunit-related protein	Regulation of beta-cell glucose-stimulated insulin secretion	DOI:10.2337/db12-1067[1]<br>DOI: 10.1007/s40618-020-01483-3[2]<br>DOI: 10.1016/j.jbc.2021.101534[3]	rs560887	2:168906638	Intron variant
GCK	7:44143213-44189439	Glucokinase	Beta-cell glucose metabolism, regulation of glucose-stimulated insulin secretion	DOI: 10.1186/s13059-023-02935-8[4]<br>DOI: 10.1016/j.tem.2022.12.007[5]<br>DOI: 10.2337/diab.39.6.647[6]	rs4607517	7:44196069	Intron variant
DGKB	7:14145049-14974858,<br>7: 15200317-15,562015	Diacylglycerol kinase beta	Cell signalling, regulation of glucose-stimulated insulin secretion	DOI: 10.1007/s00125-010-1753-5[7]<br>DOI: 10.1038/s41467-020-18581-8[8]<br>DOI: 10.1210/en.2013-1356[9]	rs2191349	7:15024684	Intergenic variant
ADCY5	3:123282296-123449090	Adenylate cyclase 5	Glucose signalling and regulation of insulin secretion, proinsulin-to-insulin conversion	DOI:10.2337/db13-1607[10]<br>DOI:10.1371/journal.pone.0023639[11]<br>DOI:10.2337/db17-0464[12]	rs11708067	3:123346931	Intron variant
CDKN2A/B	9:21967752-21995324 (A),<br>9:22002903-22009313 (B)	Cyclin-dependent kinase inhibitor 2A (p16)<br>Cyclin-dependent kinase inhibitor 2B (p15)	Beta-cell mass and function, first phase glucose-induced insulin secretion	DOI:10.1016/j.tem.2015.01.008[13]<br>DOI:10.1007/s00125-010-2038-8[14]<br>DOI:10.2337/db09-0736[15]<br>DOI:10.1152/ajpendo.00496.2010[16]	rs10811661<br><br>rs2383206	9:22134095<br><br>9:22115027	Upstream intergenic variant<br><br>Intron variant
TCF7L2	10:112950247-113167678	Transcription factor 7-like 2	Transcription factor, regulatory function of GLP-1 induced insulin response, beta-cell function	DOI: 10.1172/JCI30706[17]<br>DOI: 10.2337/db20-0573[18]<br>DOI: 10.1007/s00125-007-0753-6[19]	rs7903146	10:112998590	Intron variant

Supplementary Table 2

Diagnose codes of fragility fracture used in the Copenhagen City Heart Study and the Copenhagen General Population Study (ICD8 and ICD10) and the UK Biobank (ICD9 and ICD10) as defined by the World Health Organization's codes of International Classification of Diseases, Eight, Ninth, and Tenth Revision (ICD8, ICD9, ICD10).

Endpoint	ICD8 codes	ICD9 codes	ICD10 codes
Hip fracture	820	8200, 8202, 8208	S720-S722
Spine fracture	805.10, 805.11, 805.19	8052, 8054, 8058	S220, S221, S320, S327a, S328a, M484, M485, M495
Arm fracture (proximal humerus and wrist)	812.00, 812.01, 812.02, 812.08, 812.09, 812.19, 813.21, 813.20, 813.28, 813.29	8120, 8122, 8134, 8138	S422-S423, S525, S526, S528

Supplementary Table 3

Genetic variants used as instruments for glucose and their association with non-fasting plasma glucose in the Copenhagen studies and in the UK Biobank. β-coefficients, standard error (SE) of the β-coefficients, and p values are derived by age and sex adjusted linear regression and are shown per additional risk allele of the genetic variant.

**Gene**	**Variant**	**Copenhagen studies**		**UK Biobank**	
		**$\beta_{glucose}$ (SE)**	**P $\beta_{glucose}$**	**$\beta_{glucose}$ (SE)**	**P $\beta_{glucose}$**
G6PC2	rs560887	0.08 (0.006)	1.6×10^{-44}	0.09 (0.003)	3.4×10^{-183}
GCK	rs4607517	0.06 (0.007)	3.0×10^{-17}	0.07 (0.004)	6.5×10^{-82}
DGKB	rs2191349	0.03 (0.005)	3.3×10^{-7}	0.04 (0.003)	5.4×10^{-35}
ADCY5	rs11708067	0.04 (0.006)	2.8×10^{-11}	0.04 (0.003)	3.0×10^{-39}
TCF7L2	rs7903146	0.05 (0.006)	5.7×10^{-20}	0.07 (0.003)	3.5×10^{-98}
CDKN2A/B	rs10811661	0.03 (0.007)	7.4×10^{-7}	0.04 (0.004)	3.3×10^{-25}
CDKN2A/B	rs2383206	0.02 (0.005)	3.3×10^{-3}	0.01 (0.003)	4.4×10^{-5}
Weighted allele score		0.05 (0.002)	1.8×10^{-101}	0.05 (0.001)	1.0×10^{-300}

Supplementary Table 4

Association of the weighted allele scores (WAS) with glucose and the potential confounders body mass index, alcohol intake, smoking (in pack-years) and level of physical activity. β-coefficients, standard error (SE) of the β-coefficients, and *p* values are derived by linear regression adjusted for age and sex.

**Glucose and potential confounders**	**Copenhagen studies Glucose WAS**		**UK Biobank Glucose WAS**	
	**β (SE)**	***p* value$_β$**	**β (SE)**	***p* value$_β$**
Glucose (mmol/L)	0.05 (0.003)	2.6×10^{-95}	0.05 (0.001)	1.0×10^{-300}
Body mass index (kg/m^2)	-0.03 (0.01)	0.002	-0.02 (0.01)	0.0001
Alcohol intake (units/week)	0.00 (0.02)	0.94	-0.00 (0.00)	0.72
Pack-years, smokers	0.07 (0.04)	0.06	0.04 (0.04)	0.24
Physical activity	0.00 (0.00)	0.40	0.00 (0.00)	0.36

Supplementary Table 5.

Inverse-variance weighted regression, Mendelian randomisation (MR) Egger regression, and weighted median regression for the two-sample MR analyses using summary-level data from the Meta-Analyses of Glucose and Insulin-related traits Consortium (MAGIC) for the association between the seven genetic variants and fasting glucose and HbA_{1c}, respectively, and data on any fracture, hip fracture, and arm fracture from the Copenhagen cohorts. IVW = inverse variance weighted.

**Analysis**	**OR**	**95% CI**	***p* value**	***p* value MR Egger intercept**
Exposure: Fasting plasma glucose (MAGIC)				
Outcome: Any fragility fracture (Copenhagen studies):				
MR Egger IVW	1.36	0.89, 2.09	0.15	
MR Egger	2.10	0.85, 5.21	0.29	0.35
MR Median	1.00	0.65, 1.53	0.99	
Outcome: Hip fracture (Copenhagen studies):				
MR Egger IVW	0.90	0.53, 1.53	0.69	
MR Egger	1.04	0.32, 3.33	0.95	0.78
MR Median	0.62	0.28, 1.39	0.25	
Outcome: Arm fracture (Copenhagen studies):				
MR Egger IVW	1.50	1.03, 2.18	0.03	
MR Egger	2.28	1.05, 4.95	0.04	0.23
MR Median	2.28	1.05, 4.95	0.16	
Exposure: HbA_{1c} (MAGIC)				
Outcome: Any fragility fracture (Copenhagen studies):				
MR Egger IVW	2.47	0.95, 6.43	0.06	
MR Egger	7.63	0.99, 59.1	0.05	0.23
MR Median	2.96	1.44, 6.09	0.003	
Outcome: Hip fracture (Copenhagen studies):				
MR Egger IVW	0.90	0.53, 1.53	0.69	
MR Egger	1.97	0.08, 46.6	0.68	0.63
MR Median	0.62	0.28, 1.39	0.25	
Outcome: Arm fracture (Copenhagen studies):				
MR Egger IVW	2.79	1.12, 6.93	0.03	
MR Egger	11.19	2.26, 55.5	0.04	0.06
MR Median	3.68	1.61, 8.43	0.002	

Supplementary Figure 1.

Risk of any fragility fracture (a), hip fracture (b), and arm fracture (c) (proximal humerus and wrist) in the Copenhagen studies as a function of fasting glucose concentrations by restricted cubic spline analyses incorporated into a Cox proportional hazards model and adjusted for sex, birth year, body mass index, current smoking, physical activity level, units of alcohol consumed per week, and menopausal status for women. Fasting was defined as ≥ 6 hours since last meal and was available in 7952 individuals. Solid lines denote hazard ratios and broken lines 95% confidence intervals. The reference was set to the population median (5.2 mmol/L). Light blue area shows the distribution of glucose concentrations in the population. To convert from mmol/L to mg/dL, multiply by 18. N=number.

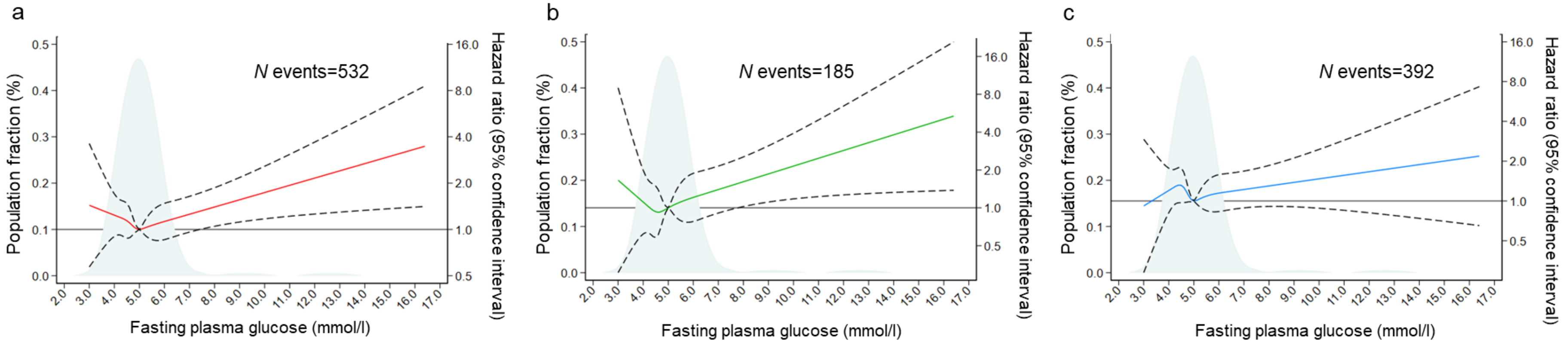

Supplementary Figure 2.

Mean concentrations of plasma non-fasting glucose (Copenhagen studies and the UK Biobank), fasting glucose (Copenhagen studies), glycated haemoglobin A1c (HbA_{1c}) (UK Biobank), and risk of type 1 and type 2 diabetes (Copenhagen studies and the UK Biobank) as a function of the glucose weighted allele scores in quintiles. Error bars denotes the standard error of the mean. Δ% denotes the percent higher concentration in non-fasting glucose, fasting glucose, and HbA_{1c} compared to the lowest quintile group. Fasting was defined ≥6 hours self-reported time since last meal. Risk of type 1 and type 2 diabetes was estimated by logistic regression adjusted for age and sex. CI= confidence interval.

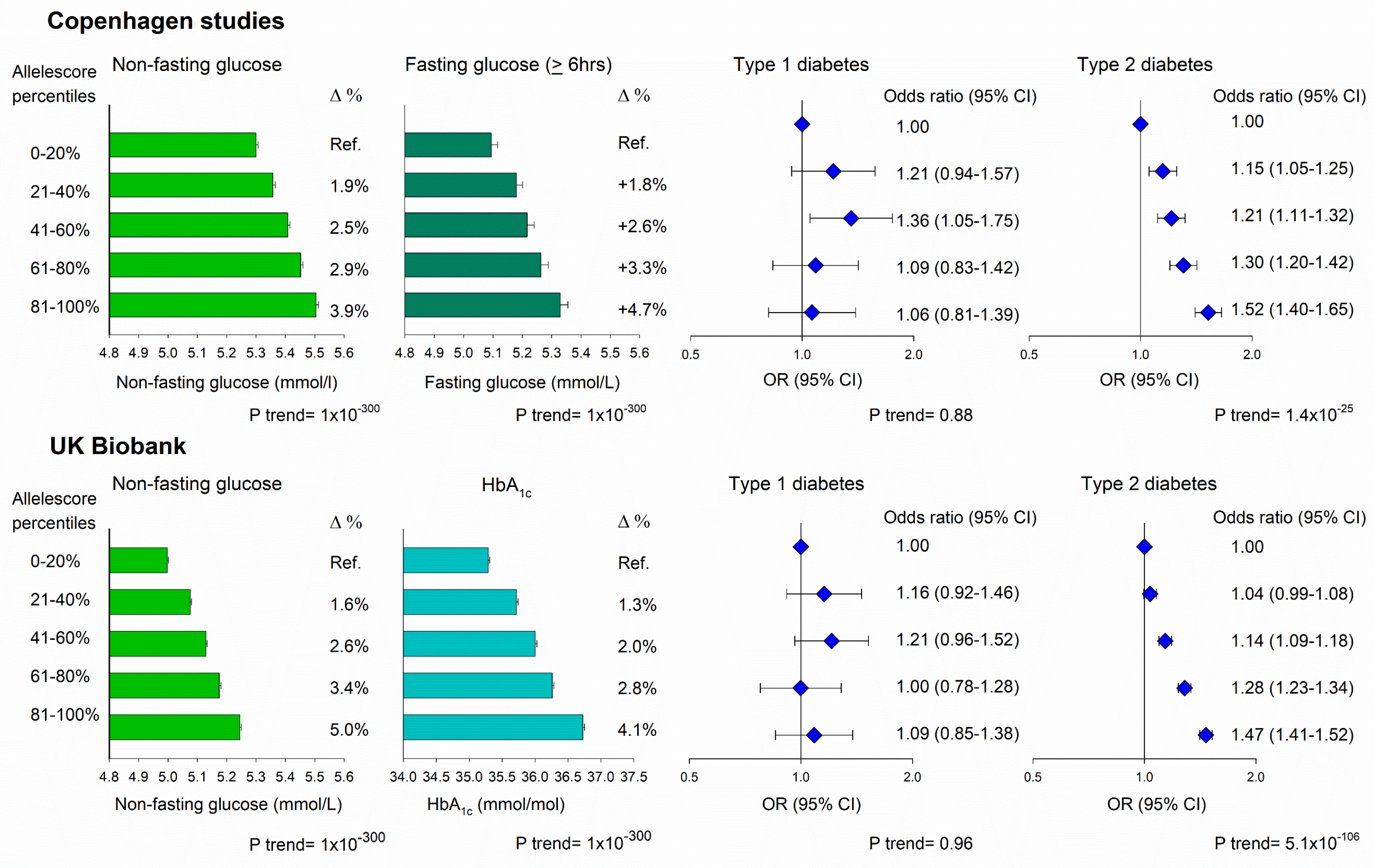

Supplementary Figure 3

Mendelian randomisation analysis of risk of any fragility fracture, hip fracture, and arm fracture (proximal humerus and wrist) in the Copenhagen studies, in UK Biobank, and in the studies combined for a 1 mmol/l higher glucose level. Estimates were derived by instrumental variable analysis adjusted for age, sex, and body mass index. *N*=number, CI=confidence interval.

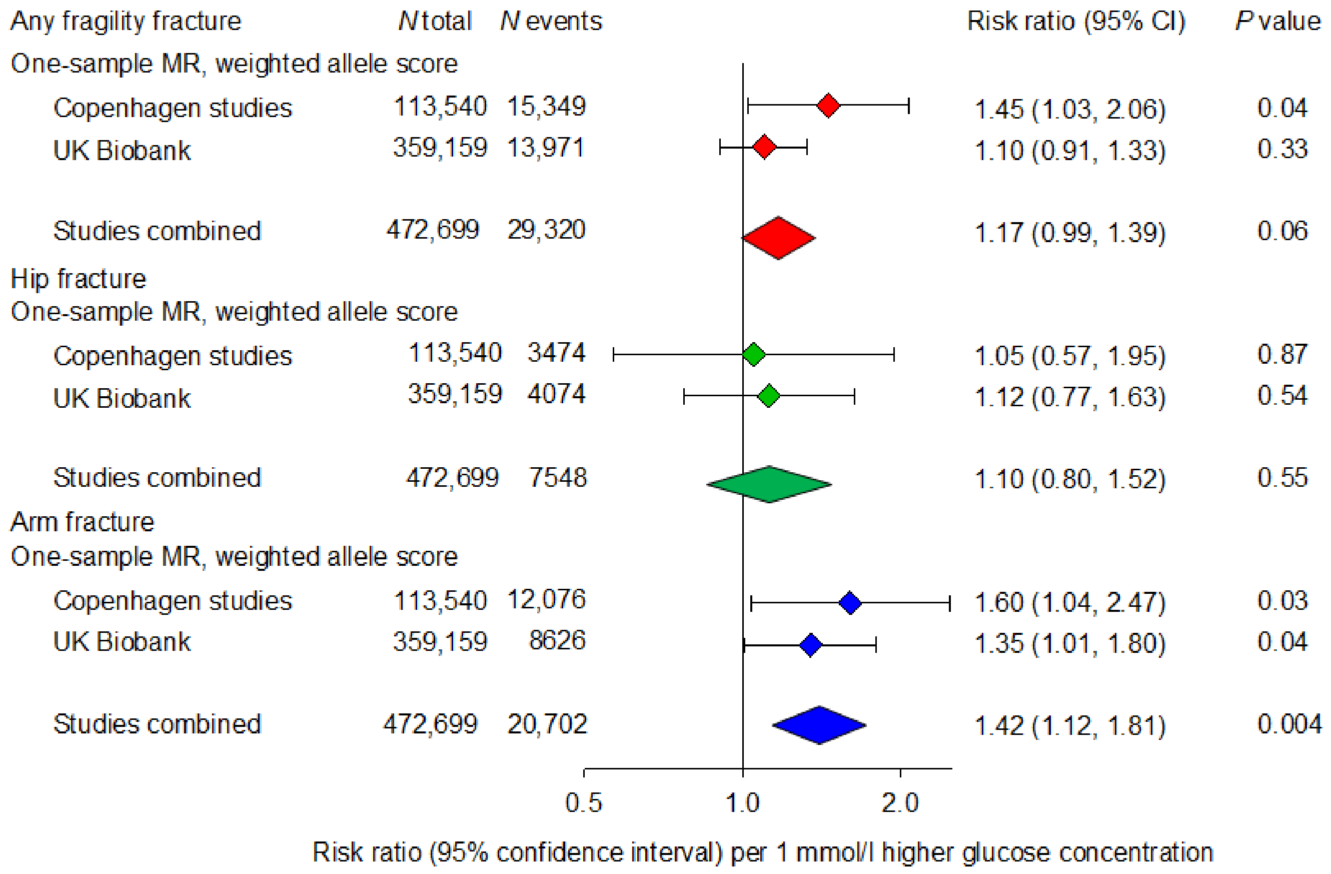

Supplementary Figure 4.

Two-sample MR analyses investigating the effect of fracture risk on fasting glucose, 2-hours post challenge glucose, HbA_{1c}, type 1, and type 2 diabetes. Publicly available data for 14 genetic variants associated with risk of fracture in the UK Biobank and replicated in the 23andMe cohorts was used as instrument and was combined with publicly available data on fasting glucose, 2-hours post-challenge glucose, and HbA1c concentrations from the Meta-Analyses of Glucose and Insulin-related traits Consortium (MAGIC), data for type 2 diabetes from the DIAMANTE consortium and data on type 1 diabetes from a GWAS by Forgetta et al. IVW = Inverse-variance weighted regression. MR = Mendelian randomization.

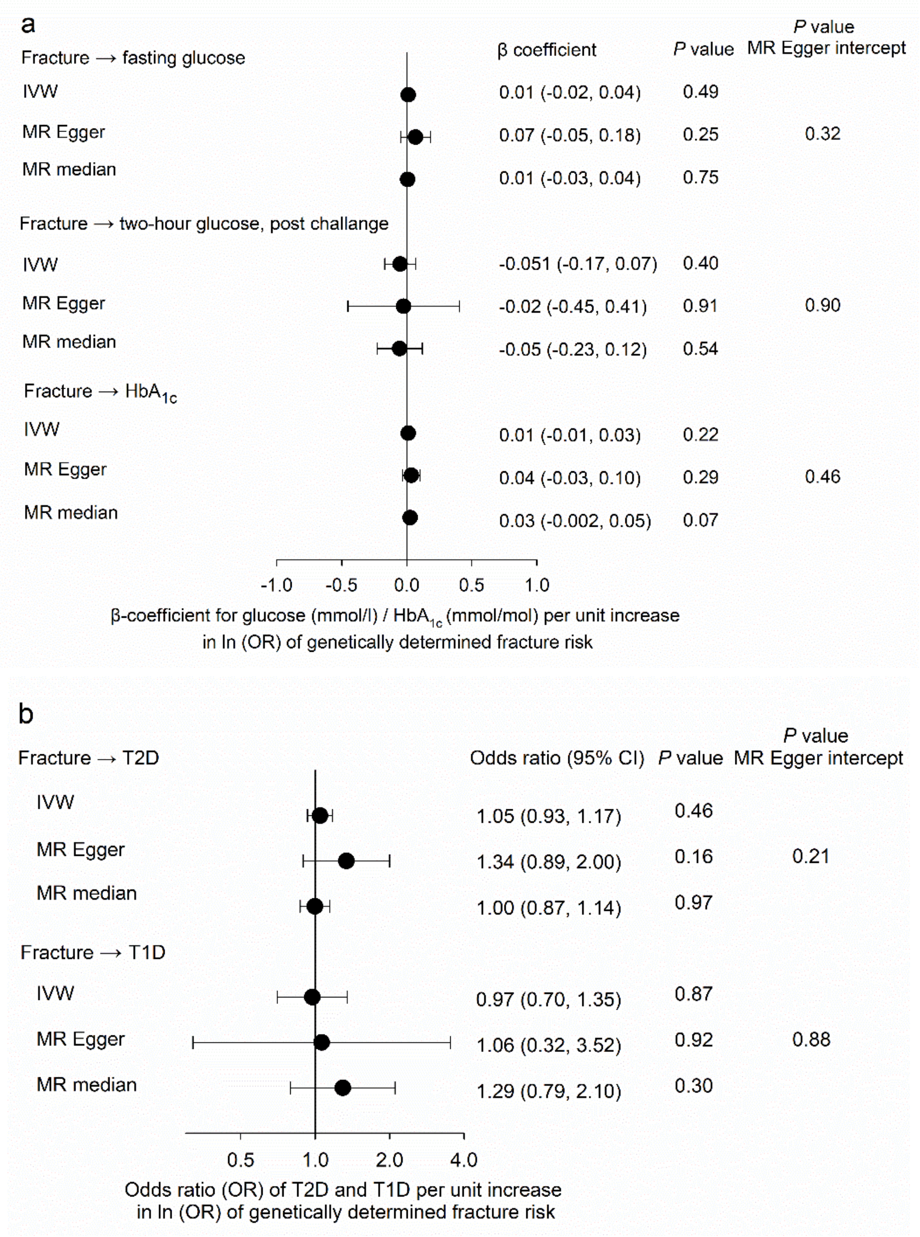

Supplementary Figure 5.

Risk of death in individuals with previous any fragility fracture in the Copenhagen studies as a function of non-fasting glucose concentrations on a continuous scale by restricted cubic spline analyses incorporated into a Cox proportional hazards model and adjusted for sex, birth year, body mass index, current smoking, physical activity level, units of alcohol consumed per week, and menopausal status for women. Solid lines denote hazard ratios and broken lines 95% confidence intervals. The reference was set to the population median (5.2 mmol/l). Light blue area shows the distribution of non-fasting glucose concentrations in the population. *N*=number.

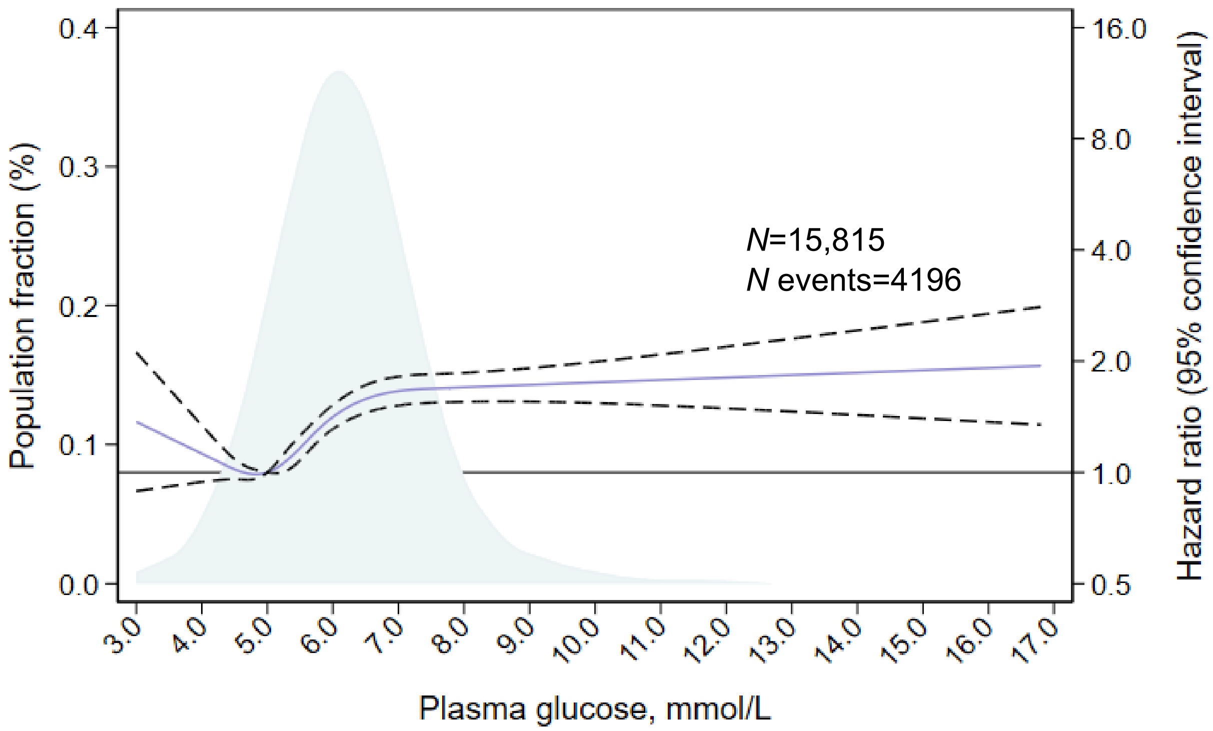

Supplementary references

1 Pound LD, Oeser JK, O'Brien TP, *et al.* G6PC2: a negative regulator of basal glucose-stimulated insulin secretion. *Diabetes* 2013; **62**: 1547–56.

2 Zusi C, Rinaldi E, Bonetti S, *et al.* Haplotypes of the genes (GCK and G6PC2) underlying the glucose/glucose-6-phosphate cycle are associated with pancreatic beta cell glucose sensitivity in patients with newly diagnosed type 2 diabetes from the VNDS study (VNDS 11). *J Endocrinol Invest* 2021; **44**: 2567–74.

3 Overway EM, Bosma KJ, Claxton DP, *et al.* Nonsynonymous single-nucleotide polymorphisms in the G6PC2 gene affect protein expression, enzyme activity, and fasting blood glucose. *J Biol Chem* 2022; **298**: 101534.

4 Gersing S, Cagiada M, Gebbia M, *et al.* A comprehensive map of human glucokinase variant activity. *Genome Biol* 2023; **24**: 97.

5 Ashcroft FM, Lloyd M, Haythorne EA. Glucokinase activity in diabetes: too much of a good thing? *Trends Endocrinol Metab* 2023; **34**: 119–30.

6 Matschinsky FM. Glucokinase as Glucose Sensor and Metabolic Signal Generator in Pancreatic β-Cells and Hepatocytes. *Diabetes* 1990; **39**: 647–52.

7 Boesgaard TW, Grarup N, Jørgensen T, *et al.* Variants at DGKB/TMEM195, ADRA2A, GLIS3 and C2CD4B loci are associated with reduced glucose-stimulated beta cell function in middle-aged Danish people. *Diabetologia* 2010; **53**: 1647–55.

8 Viñuela A, Varshney A, van de Bunt M, *et al.* Genetic variant effects on gene expression in human pancreatic islets and their implications for T2D. *Nat Commun* 2020; **11**: 4912.

9 Kurohane Kaneko Y, Kobayashi Y, Motoki K, *et al.* Depression of Type I Diacylglycerol Kinases in Pancreatic β-Cells From Male Mice Results in Impaired Insulin Secretion. *Endocrinology* 2013; **154**: 4089–98.

10 Hodson DJ, Mitchell RK, Marselli L, *et al.* ADCY5 Couples Glucose to Insulin Secretion in Human Islets. *Diabetes* 2014; **63**: 3009–21.

11 Wagner R, Dudziak K, Herzberg-Schäfer SA, *et al.* Glucose-raising genetic variants in MADD and ADCY5 impair conversion of proinsulin to insulin. *PLoS One* 2011; **6**: e23639.

12 Roman TS, Cannon ME, Vadlamudi S, *et al.* A Type 2 Diabetes-Associated Functional Regulatory Variant in a Pancreatic Islet Enhancer at the ADCY5 Locus. *Diabetes* 2017; **66**: 2521–30.

13 Hannou SA, Wouters K, Paumelle R, Staels B. Functional genomics of the CDKN2A/B locus in cardiovascular and metabolic disease: what have we learned from GWASs? *Trends Endocrinol Metab* 2015; **26**: 176–84.

14 Hribal ML, Presta I, Procopio T, *et al.* Glucose tolerance, insulin sensitivity and insulin release in European non-diabetic carriers of a polymorphism upstream of CDKN2A and CDKN2B. *Diabetologia* 2011; **54**: 795–802.

15 't Hart LM, Simonis-Bik AM, Nijpels G, *et al.* Combined Risk Allele Score of Eight Type 2 Diabetes Genes Is Associated With Reduced First-Phase Glucose-Stimulated Insulin

Secretion During Hyperglycemic Clamps. *Diabetes* 2010; **59**: 287–92.

16 Köhler CU, Olewinski M, Tannapfel A, Schmidt WE, Fritsch H, Meier JJ. Cell cycle control of β-cell replication in the prenatal and postnatal human pancreas. *Am J Physiol Metab* 2011; **300**: E221–30.

17 Lyssenko V, Lupi R, Marchetti P, *et al.* Mechanisms by which common variants in the TCF7L2 gene increase risk of type 2 diabetes. *J Clin Invest* 2007; **117**: 2155–63.

18 del Bosque-Plata L, Martínez-Martínez E, Espinoza-Camacho MÁ, Gragnoli C. The Role of TCF7L2 in Type 2 Diabetes. *Diabetes* 2021; **70**: 1220–8.

19 Schäfer SA, Tschritter O, Machicao F, *et al.* Impaired glucagon-like peptide-1-induced insulin secretion in carriers of transcription factor 7-like 2 (TCF7L2) gene polymorphisms. *Diabetologia* 2007; **50**: 2443–50.
